# Genotypic Diversity of* Mycobacterium tuberculosis* Clinical Isolates in the Multiethnic Area of the Xinjiang Uygur Autonomous Region in China

**DOI:** 10.1155/2017/3179535

**Published:** 2017-02-28

**Authors:** Jie Liu, Junlian Li, Jiao Liu, Xiuqin Zhao, Lulu Lian, Haican Liu, Bing Lu, Qin Yu, Jingrui Zhang, Yingcheng Qi, Kanglin Wan

**Affiliations:** ^1^National Institute for Communicable Disease Control and Prevention, Chinese Center for Disease Control and Prevention, State Key Laboratory for Infectious Disease Prevention and Control, Beijing 102206, China; ^2^Collaborative Innovation Center for Diagnosis and Treatment of Infectious Diseases, Hangzhou 310003, China; ^3^Chaoyang Center for Disease Control and Prevention, Beijing, 100021, China; ^4^The Chest Hospital of Xinjiang Uyghur Autonomous Region, Ürümqi 830001, China

## Abstract

*Objectives.* We studied the genetic diversity of clinical isolates from patients with tuberculosis in the multiethnic area of Xinjiang autonomous region in China. A total of 311 clinical* M. tuberculosis* isolates were collected in 2006 and 2011 and genotyped by two genotyping methods. All isolates were grouped into 68 distinct spoligotypes using the spoligotyping method. The Beijing family was dominant, followed by T1 and CAS. MIRU-VNTR results showed that a total of 195 different VNTR types were identified. Ten of the 15 loci were highly or moderately discriminant according to their HGDI scores, and 13 loci had good discriminatory power in non-Beijing family strains, whereas only two loci had good discriminatory power in Beijing family strains. Chi-square tests demonstrated that there were no correlations between four characteristics (sex, age, type of case, and treatment history) and the Beijing family. In summary, Beijing family strains were predominant in Xinjiang, and the VNTR-15_China_ locus-set was suitable for genotyping all Xinjiang strains, but not for the Beijing family strains. Thus, these data suggested that different genotype distributions may exist in different regions; MLVA locus-sets should be adjusted accordingly, with newly added loci to increase resolution if necessary.

## 1. Introduction

Tuberculosis (TB) is a severe chronic infectious disease caused by* Mycobacterium tuberculosis* (*M. Tuberculosis*), which remains prevalent despite intense global efforts to control and eliminate this disease. In 2014, 9.6 million people were estimated to have contracted TB, and 1.4 million TB-related deaths occurred [1]. Of the 22 countries accounting for 79% of the world's burden of TB, China is ranked second and has the highest absolute number of cases annually worldwide [2].

The Beijing family genotype of* M. tuberculosis* was first described in 1995 by Van Soolingen, and 86% of isolates from Beijing, China, were found to have this Beijing family genotype [3]. However, the distribution of* M. tuberculosis* and the proportions of Beijing family isolates in Xinjiang are unclear.

The Xinjiang Uygur autonomous region is located in northwestern China, surrounded by India, Russia, Pakistan, Mongolia, and other countries, and covers one-sixth of the land area of China (a total of 1.66 million km^2^). Thus, this province is the largest province in China and has the longest land borderline with neighboring countries. In 2010, the population of this region was 22 million, and the region was multiethnic, comprised of Uygur, Kazak, Hui, and other ethnic minorities. The minority population in this region accounts for approximately 60.5% of the population. The Xinjiang autonomous region has a high TB burden and TB prevention and control measures are needed owing to the unique geographical location and complex ethnic composition of this region.

In this study, we used spacer-oligonucleotide typing (spoligotyping) and multiple locus variable number tandem repeat (VNTR) analysis (MLVA), which both employ polymerase chain reaction- (PCR-) based genotyping technology, to characterize* M. tuberculosis* genotypes circulating in the Xinjiang Uygur autonomous region and to explore whether there were relationships between the spread of Beijing family strains and patient characteristics, including sex, age, type of case, and treatment history. In addition, we evaluated the discriminatory power of 15-loci-set MLVA (VNTR-15_China_) [4] to characterize the strains from the Xinjiang Uygur autonomous region.

## 2. Materials and Methods

### 2.1. *M. tuberculosis* Strains

A total of 311* M. tuberculosis* isolates were obtained from sputum samples collected from patients with pulmonary TB in 2006 and 2011 at the Provincial Tuberculosis Hospital of Xinjiang. All patients with TB were diagnosed based on the national guidelines of China. Demographic, epidemiological, and clinical data were obtained from the medical records of all patients; these data included sex, age, present address, diagnosis results, drug susceptibility test results, previous TB history, symptoms, and associated medical data from local doctors working in the hospital using uniform epidemiological investigation methods. For the 311* M. tuberculosis* strains, detailed information is provided in Table 1.

### 2.2. DNA Sample Preparation

Bacteria were isolated and inoculated on Löwenstein-Jensen (L-J) medium. Culture was performed for all samples and the bacteria were kept at the National Laboratory of TB, ICDC, China CDC, Beijing, China.* Mycobacterial* genomic DNA was extracted from mycobacterial colonies grown on L-J medium by resuspending one loopful of mycobacterial colonies in 200 *μ*L TE buffer (10 mM Tris-HCl, 1 mM ethylenediaminetetraacetic acid [EDTA]) and was incubated at 85°C for 30 min. The supernatant containing the DNA was then collected by centrifugation at 12,000 rpm for 5 min and stored at −20°C for further use.

### 2.3. Spoligotyping

Spoligotyping involves PCR-based amplification of the whole CRISPR region with the primers DRa and DRb (DRa: 5′-GGT TTT GGG TCT GAC GAC-3′, DRb: 5′-CCG AGA GGG GAC GGA AAC-3′), followed by hybridization of the amplified DNA to a set of 43 spacer-oligonucleotides probes corresponding to each spacer, covalently linked to a membrane. Because clinical isolates vary in the nature of spacer sequences, the spoligotype patterns obtained were strain specific. Detailed procedures were described previously [5]. The results were analyzed using BioNumerics software (version 5.0). The Beijing genotype here was any isolate missing spacers 1 to 34, with at least three spacers from 35 to 43 [5, 6].

### 2.4. MLVA Typing

In addition to spoligotyping, the MLVA typing method based on VNTR-15_China_ was also carried out, described by Wan et al. [7], for most of the isolates collected from more than 14 provinces in China [4, 7, 8]. The discrimination of the locus combination was calculated using the Hunter-Gaston discriminatory index (HDGI), calculated using the following formula [9]:

(1) $$\begin{array}{c} HDGI=1-\left[\;\frac{1}{N\left(N-1\right)}\overset{s}{\underset{j=1}{\sum }}nj\left(\;nj-1\;\right)\;\right], \end{array}$$

where* N* is the total number of isolates in the typing method,* s* is the number of distinct patterns discriminated by MIRU-VNTR, and* nj* is the number of isolates belonging to the* j*th pattern.

### 2.5. Data Analysis

Genotype results were entered in binary format into a Microsoft Excel spreadsheet, as shown in Table S1. The patterns were established based on clusters generated in BioNumerics software version 5.0 (Applied Maths, Sint-Martens-Latem, Belgium). Spoligotypes were designated according to the updated version of the international spoligotype database SITVIT2 (http://www.pasteur-guadeloupe.fr:8081/SITVITDemo). Statistical data were analyzed using the Chi-square tests. All statistical tests were two-sided, and differences with *P* values of less than 0.05 were considered significant. Statistical analyses were carried out using SPSS software 19.0 (SPSS Inc., Chicago, IL, USA).

## 3. Results

We collected 311 isolates from patients clinically diagnosed with pulmonary TB in this study in 2006 and 2011. Patient demographics are shown in Table 1.

### 3.1. Spoligotyping Analysis

Spoligotyping results showed that the 311 isolates in this study could be grouped into 68 distinct spoligotypes; 54 strains represented a single isolate, whereas the other 257 isolates were grouped into 14 clusters containing from two to 212 isolates, with a cluster rate of 78.14%. According to SITVIT2, 271 (87.13%) strains were classified into 29 shared international types (SITs), and 40 (12.87%) strains were found to not have SIT number. In the seven families (Beijing, T, Haarlem, CAS, LAM9, MANU2, and U), MANU2 and LAM9 had only one isolate, and the other families had two or more strains. The Beijing family was the dominant genotype, with 224 isolates (72.03%), followed by T (20, 6.43%) and CAS (12, 3.86%; Table 2).

A total of 311 strains were collected in the Xinjiang area; 171 strains were collected in 2006, and 140 strains were collected in 2011. The genotype distribution of strains in different years is shown in Table 3. Compared with that in 2006, the proportions of strains belonging to the Beijing and T families increased in 2011 and the proportion of new genotypes decreased.

### 3.2. MLVA

A total of 195 different VNTR types were identified among the 311 strains. One hundred fifty-four (49.52%) strains were unique, and 157 (50.48%) strains could be grouped into 41 clusters, containing from two to 31 strains, with a cluster rate of 37.30%.

Next, we evaluated the discriminating ability of MIRU-VNTR loci based on HGDI scores classified as high (>0.6), moderate (0.3 to 0.6), and poor (<0.3) [10]. For the genotyping of all 311 strains, HGDI scores were calculated from 0.778 to 0.069 among 15 MIRU-VNTR loci. MIRU26 (0.778) and Mtub21 (0.691) had high discriminating ability. Additionally, four loci (ETRB, ETRC, ETRD, and MIRU27) had poor discrimination ability (HGDI < 0.3), and MIRU23 (0.069) showed almost negligible diversity (HGDI < 0.1; Table 4). Thirteen loci had good discriminatory power in non-Beijing family strains, whereas only two loci had good discriminatory power in Beijing family strains.

### 3.3. Comparisons between Spoligotyping and 15-Loci MLVA

As shown in Figure S1 (in Supplementary Material available online at https://doi.org/10.1155/2017/3179535), there was good agreement between the two methods; only two non-Beijing family strains were clustered with Beijing family strains when we used 15-loci-set MLVA. This may be due to the presence of two independent strains in some clinical samples. Eight spoligotype variants of the Beijing family were detected in the 224 Beijing family strains, and the HGDI score was 0.103. Moreover, these strains were distributed into 119 genotypes by MLVA, with an HGDI score of 0.986 confirming that VNTR-15_China_ MLVA was more suitable for typing Beijing strains than spoligotyping.

In 224 Beijing family strains, 89 (39.73%) strains were unique, and 135 (60.27%) strains could be grouped into 33 clusters, with a cluster rate of 45.53%. Sixty-five (74.71%) strains were unique in a total of 87 non-Beijing family strains, and 22 (25.29%) strains could be grouped into eight clusters, with a cluster rate of 16.09%.

### 3.4. Relationship between Beijing Family Genotypes and Strain Characteristics

Four factors associated with TB, that is, sex, age, case type, and treatment history, were included in this study. We found that there were no correlations between Beijing family genotypes and the four factors associated with TB (*P* > 0.05 for all; Table 5).

## 4. Discussion

Efficient disease control can be achieved using epidemiological surveillance systems to accurately monitor epidemic trends at the regional and global levels [11]. Genotyping of* M. tuberculosis* plays an important role in epidemiological studies [12], and genetic analyses have suggested that* M. tuberculosis *exhibits substantial genetic variations [13], such as large sequence polymorphisms (LSPs) [14], single-nucleotide polymorphisms (SNPs), variable numbers and locations of insertion element (IS)* 6110 *[15], and VNTRs [16], all of which have been commonly employed in molecular epidemiology. However, because a single genotyping method cannot define all unique isolates, the current studies undertaken require various strategies to increase the power of strain differentiation [17, 18]. In this study, by the comparison of MLVA and spoligotyping methods, we confirmed that the VNTR-15_China_ loci set was suitable for typing strains in China.

Spoligotyping results showed that the cluster rate was 78.14%. In the MLVA results, we found that patients infected with a Beijing family strain were more likely to be clustered than patients who were infected with a non-Beijing family strain. The MLVA-VNTR cluster case, which was produced by the same source of infection spread, had the same genotype in the short term. Moreover, in this study, the cluster rate of the MLVA-VNTR was 37.30%, indicating that 37.30% of patients may have acquired infections from the recent spread. However, this analysis was likely to have overestimated the recent spread. A study showed that, during the course of evolution, some strains at great distances may form the same VNTR genotype [19]. This is a limitation of this method, which we called VNTR homoplasy [20]. The genetic distance of Beijing family strains was relatively close; thus, it may be more likely for these strains to form the same VNTR genotype during evolution. In addition, Beijing family strains are quite abundant, leading to a higher cluster rate.

*M. tuberculosis* Beijing family strains are the most prevalent strains in China [8]. In this study, according to spoligotyping results, the Beijing genotype was also predominant in the Xinjiang region; however, the proportion of the Beijing genotype in Xinjiang, which is located in northwestern China, was lower than that in other provinces in northern China [8, 21–24], but higher than those of the areas in southern China [25–30]. These results could be explained by the particular features of the regions, including geographic, climatic, or ethnic differences [8]. Beijing family strains were also found to be dominant in some East Asian countries, such as South Korea (97.1%) [31], Thailand (44%) [32], and Vietnam (53%) [33, 34]. Moreover, within the past decade, the molecular epidemiological data from some areas have revealed that the Beijing family genotype is widespread around the world [35].

In our study, in addition to Beijing family strains, we also detected strains belonging to other families, such as T1, U, H4, T2, MANU2, and LAM9. One interesting finding in this study was that 12 (3.86%) strains tested belonged to the CAS family, which has only been found in Tibet [23] and Xinjiang in China. All CAS family strains originated from patients of Tibetan and Uyghur ethnicity before 2010. This finding suggested that the CAS family may have associated with the patient's ethnic groups and regional distribution. Moreover, Tibet and Xinjiang share geographic borders with India, where the CAS family is dominant [36]. This family of strains may also be transported by trade, tourism, or migration from India. When we first found CAS family strains in 2006, all strains were isolated from Uygur individuals. Now, we detected one CAS strain from a Han ethnic patient in 2011 and found CAS strains isolated from a Han ethnic patient in Gansu, 2011 [4]. These findings suggested that CAS family strains have the potential to spread inland. The LAM family was also found in Jiangsu province and Taiwan in China [26, 30], although this family of strains is predominantly prevalent in South America [37] and West Africa [38].

The spoligotyping typing method has the advantage of identification of Beijing family strains, but with lower ability to distinguish among strains in comprehensive analysis. Thus, we performed spoligotyping in combination with MLVA and found a general HGDI score of 0.986 for this combined method. Ten of the 15 loci were highly or moderately discriminatory according to their HGDI scores, and 13 loci had good discriminatory power in non-Beijing family strains, whereas only two loci had good discriminatory power in Beijing family strains. Thus, some non-Beijing family strains may have spread from neighboring countries, with a variety of genotypes, and most loci may have permitted good discrimination of non-Beijing strains. Additionally, since the 1950s, many immigrants have entered China owing to changes to the national migration plan and individuals have migrated from some provinces to Xinjiang [39]; some of these immigrants may have brought tuberculosis, with the Beijing family strains remaining the predominant strains. Accordingly, the proportion of Beijing family strains has increased in these patients, although little variation has occurred owing to the recent spread of infection, resulting in poor discrimination ability of these loci. This result shows the limitations of the low discrimination power of some loci for epidemiology studies in such a high homogeneity group, Beijing family. In addition, because non-Beijing family strains can be subdivided into numerous lineages, they show good discriminatory power. Therefore, conclusions on the discriminatory power in Beijing/non-Beijing family strains need to be drawn carefully.

Based on the potential for worldwide dissemination of the* M. tuberculosis* Beijing family genotype, particularly the high infection rate in China, we aimed to determine the cause of this problem. We attempted to combine Beijing family strains with demographic data in order to investigate the correlations between the Beijing family genotype and the general characteristics of patients with TB. The results showed that there were no significant correlations of sex, age, treatment history, and case type with the distribution of Beijing family strains; thus, we can speculate that these four factors were likely not correlated with the prevalence of Beijing family strains. These results were similar to those of previous studies [16, 40, 41]. Therefore, we suggest that there may be other factors promoting the transmission of the* M. tuberculosis* Beijing family. Some researchers speculated that the long-term* M. bovis* BCG vaccine may be one of the selective forces implicated in the successful spread of the Beijing genotype [42, 43] and that drug resistance (particularly multiple drug resistance) may be a factor enhancing the spread of this family [44]. These two hypotheses are still controversial because they have not been investigated sufficiently. Thus, although other factors may also promote the spread of the Beijing family genotype of* M. tuberculosis*, additional studies are required to confirm this assertion.

In summary, this is the first report applying spoligotyping in combination with VNTR-15_China_ loci-set MLVA technology for genotyping of TB strains in the Xinjiang autonomous region of China. Our results showed that Beijing family strains were predominant in Xinjiang and that the VNTR-15_China_ loci set was suitable for genotyping all Xinjiang strains, but not all Beijing family strains. Thus, these data suggested that different genotype distributions may exist in different regions; MLVA locus sets should be adjusted accordingly with newly added loci to increase resolution if necessary.

## Supplementary Material

Table S1 is a Fifteen-loci MIRU-VNTR and spoligotyping profile which providing typing data by the 15 loci MLVA, and spoligotyping of the 311 isolates.
Figure S1 is genotyping of 311 M. tuberculosis isolates with VNTR15-China and Spoligotyping. The clustering was based on the analysis performed using BioNumerics 6.5 to compare these two genotyping methods. From left to right: 1) UPGMA dendrogram generated by VNTR15-China 2) poligotyping patterns 3)the repeat number in each VNTR-loci 4) strain number, and 5) the lineage strain belongs to.

## Figures and Tables

**Table 1:** Patient demographics.

Characteristics	Number (%) of strains		
	2006	2011	Total
*Total*	171 (54.98)	140 (45.02)	311 (100)
*Sex*			
Male	100 (32.15)	74 (23.80)	174 (55.95)
Female	71 (22.83)	65 (20.90)	136 (43.73)
Unknown	0	1 (0.32)	1 (0.32)
*Age group*			
≤20	9 (2.89)	14 (4.50)	23 (7.39)
~20	79 (25.41)	55 (17.68)	134 (43.09)
~40	41 (13.18)	40 (12.87)	81 (26.05)
≥60	37 (11.90)	30 (9.64)	67 (21.54)
Unknown	5 (1.61)	1 (0.32)	6 (1.93)
*Type of case*			
Relapse	42 (13.50)	25 (8.04)	67 (21.54)
New case	114 (36.66)	19 (6.11)	133 (42.77)
Unknown	15 (4.82)	96 (30.87)	111 (35.69)
*Treatment history*			
Yes	62 (19.94)	31 (9.96)	93 (29.90)
No	81 (26.05)	10 (3.21)	91 (29.26)
Unknown	28 (9.00)	99 (31.84)	127 (40.84)

**Table 2:** Spoligotypes of prevalent clades by SITVIT2 in this study.

Number	Spoligotype^a^	SIT	Family^b^	*N* ^c^
1	□□□□□□□□□□□□□□□□□□□□□□□□□□□□□□□□□□■■■■■■■■■	1	Beijing	212
2	□□□□□□□□□□□□□□□□□□□□□□□□□□□□□□□□□□■■■■■□■■■	190	Beijing	5
3	□□□□□□□□□□□□□□□□□□□□□□□□□□□□□□□□□□■■■□■■■■■	632	Beijing	2
4	□□□□□□□□□□□□□□□□□□□□□□□□□□□□□□□□□□□□□□□□■■■	585	Beijing-like	1
5	□□□□□□□□□□□□□□□□□□□□□□□□□□□□□□□□□□■□□□□□■■■	—	Beijing-like	1
6	□□□□□□□□□□□□□□□□□□□□□□□□□□□□□□□□□□□□□□□■■■■	—	Beijing-like	1
7	□□□□□□□□□□□□□□□□□□□□□□□□□□□□□□□□□□■■■■■■■□■	1674	Beijing	1
8	□□□□□□□□□□□□□□□□□□□□□□□□□□□□□□□□□□■■■■■■■□□	—	Beijing-like	1
9	■■■■■■■■■■■■■■■■■■■■■■■■■■■■■■■■□□□□■■■□■■■	52	T2	5
10	■■■■■■■■■■■■■■■■■■■■■■■■■■■■■■■■□□□□■■■■■■■	53	T1	9
11	■■□■■■■■■■■■■■■■■■■■■■■■■■■■■■■■□□□□■■■■■■■	196	T1	1
12	■■■■□■■■■■■■■■□■■■■■■■■■■■■■■■■■□□□□■■■■■■■	1221	T1	1
13	■■■■□■■■■■■■■■■■■■■■■■■■■■■■■■■■□□□□■■■□■■■	153	T2	1
14	■■■■■■■■■■■■■■□■■■■■■■■■■■■■■■■■□□□□■■■■■■■	76	T2	2
15	■■■■■■■■■■■■■■■■■■■■■□■■■■■■■■■■□□□□■■■■■■■	86	T1	1
16	■■■■■■■■■■■■■■■■■■■■□□□□■■■■■■■■□□□□■■■■■■■	42	LAM9	1
17	■□■■■■■■■■■■■■■■■■■■■■■■■■■■■■■■□□■■■■■■■■■	1096	MANU2	1
18	■■■■■■■■■■■■■■■■■■■■■■■■■■■■■■□■□□□□■■■■■■■	50	H3	1
19	■□■■■■■■■■■■■■■■■■■■■■■■■■■■□□□■□□□□■■■■■■■	127	H4	7
20	■■■■■■■■■■■■□■■■■■■■■■■■■■■■□□□■□□□□■■■■■■■	35	H4	2
21	■■■□□□□■■□■■■■■■■■■■■■□□□□□□□□□□□□■■□□■■■■■	1314	CAS1-DELHI	1
22	■■■□□□□■■■■■■■■■■■■■■■□□□□□□□□□□□□■■■■■■■■■	26	CAS1-DELHI	2
23	■■■□□□□■■■■■■■■■■■■■■■□□□□□□□□□□□□■■□□■■■■■	29	CAS1-DELHI	3
24	■■■□□□□■■■■■■■■■■□□□□□□□□□□□□□□□□□□□□□□□□■■	1777	CAS1-DELHI	1
25	■■■□□□□□■■■■■■■■■■■■■■□□□□□□□□□□□□□□■■■■■■■	—	CAS1-DELHI	1
26	■■■□□□□■■■■■■■■■■■■■■■□□□□□□□□□□□□□□■■■■■■■	357	CAS	2
27	■■■□□□□□■■■■■■■■■■■■■■□□□□□□□□□□□□■■■■■■■■■	1551	CAS	1
28	■■■■■■■■■■■■■■■■■■■■■■■■■■■■■■■■■□□□■■■■■■■	1378	CAS	1
29	■■■■■■■■■■■■■■■■■■■■■■■■■■■■■■■□□□□□□□□□■■■	—	U	1
30	■■■■■■■■■■■■■■■■■■■■■■■■■■■■■■■■□□□□□■■■■■■	240	U	2
31	■■■■■■■□■■■■■■■■■■■■□■■■■■■■■■■■□□□□■■■■■■■	—	U	1
32	■■■■■■□□■■■■■■■■■■■■□■■■■■■■■■■■□□□□■■■■■■■	—	U	1
33	■■■■■■■■■■■■■■■■■■■■■■■■■■■■■■■□□□□□□■■■■■■	519	U	1
34	■■■□□□□■■■■■■■■■■■■■■■□□■■■■■■■■■□□□□■■■■■■	27	U	1
35	■■■■■■■■■■■■□■■■■■■■■■■■□□□□□□□□□□□□□□□□□□□	56	U	2

^a^Presence of spacer (■); absence of spacer (□).

^b^Representing spoligotype families as assigned in SITVIT2.

^c^ *N*: number of strains.

**Table 3:** Genotype distributions of clinical isolates in different years.

Family^a^	2006		2011	
	*N* ^b^	%	*N*	%
Beijing	117	68.42	107	76.43
T	9	5.26	11	7.86
Haarlem	7	4.09	3	2.14
CAS	6	3.51	6	4.29
LAM9	0	0	1	0.71
MANU2	1	0.58	0	0
U	7	4.09	2	1.43
New	24	14.04	10	7.14

Total	171	100	140	100

^a^Representing spoligotype families as assigned in SITVIT2.

^b^ *N*: number of strains.

**Table 4:** HGDI scores of the different MIRU-VNTR loci.

	All strains	Beijing family strains	Non-Beijing family strains
MIRU26	0.778	0.717	0.766
Mtub21	0.691	0.526	0.795
MIRU10	0.498	0.217	0.746
ETRE	0.469	0.187	0.500
MIRU40	0.415	0.217	0.711
ETRA	0.405	0.205	0.615
MIRU16	0.402	0.207	0.703
Mtub30	0.400	0.062	0.408
MIRU39	0.385	0.096	0.464
Mtub39	0.306	0.139	0.618
ETRC	0.243	0.130	0.435
ETRB	0.187	0.027	0.443
ETRD	0.129	0.045	0.315
MIRU27	0.122	0.045	0.288
MIRU23	0.069	0.045	0.127

**Table 5:** Statistical analysis of the relationship between *M. tuberculosis* Beijing family and the factors associated with TB.

Factors	Total	Beijing family (%)	Non-Beijing family	*χ* ^2^	*P*
Sex					
Male	174	123 (70.69)	51	0.070	0.791
Female	136	98 (72.06)	38		
Age group					
≤20	23	19 (82.61)	4	3.165	0.367
~20	134	97 (72.39)	37		
~40	81	58 (71.60)	23		
≥60	67	43 (64.18)	24		
Type of case					
Relapse	67	51 (76.12)	16	3.412	0.065
New case	133	84 (63.16)	49		
Treatment history					
Yes	93	62 (66.67)	31	0.045	0.832
No	91	62 (68.13)	29		
