# Supplementary material for: Genotypic Diversity of Mycobacterium tuberculosis Clinical Isolates in the Multiethnic Area of the Xinjiang Uygur Autonomous Region in China
Source: Biomed Res Int. 2017 Feb 28;2017:3179535. doi: 10.1155/2017/3179535 (PMC5350424; doi:10.1155/2017/3179535)
Supplement: Supplementary file 1 — Table S1 is a Fifteen-loci MIRU-VNTR and spoligotyping profile which providing typing data by the 15 loci MLVA, and spoligotyping of the 311 isolates. Figure S1 is genotyping of 311 M. tuberculosis isolates with VNTR15-China and Spoligotyping. The clustering was based on the analysis performed using BioNumerics 6.5 to compare these two genotyping methods. From left to right: 1) UPGMA dendrogram generated by VNTR15-China 2) poligotyping patterns 3)the repeat number in each VNTR-loci 4) strain number, and 5) the lineage strain belongs to. [file 3179535.f1.pdf]

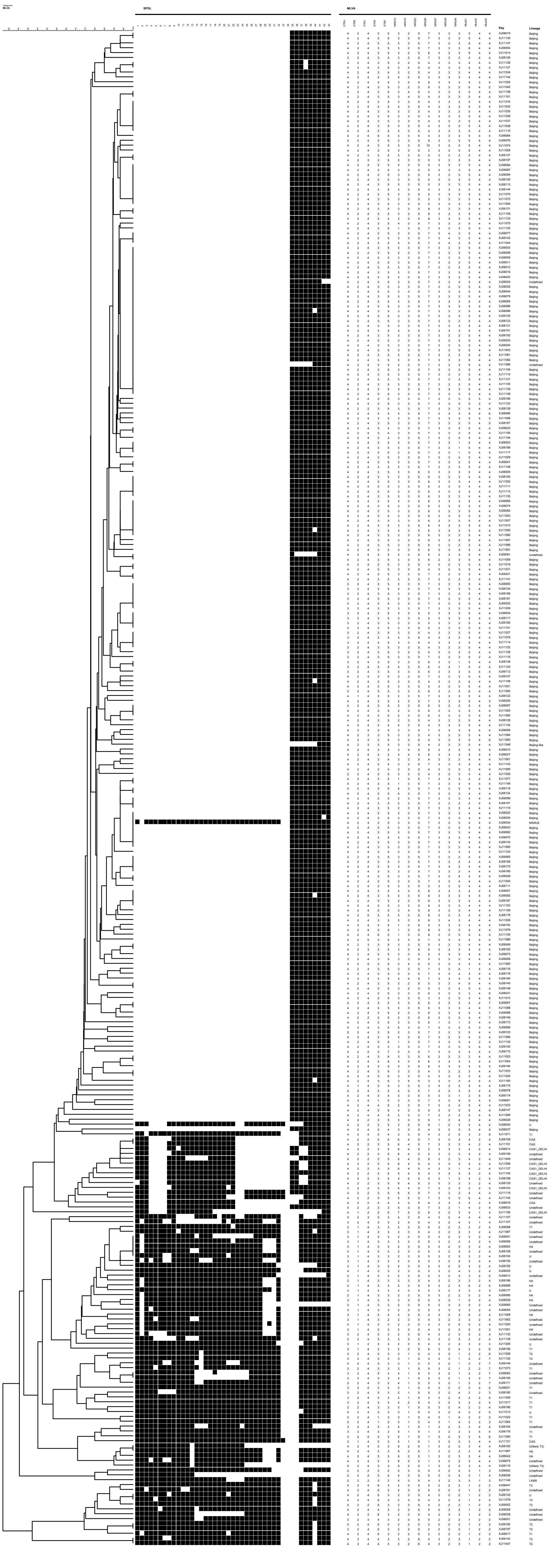

Figure S1

Table S1

Strain N	1	2	3	4	5	6	7	8	9
H37Rv	1	1	1	1	1	1	1	1	1
BCG	1	1	0	1	1	1	1	1	0
Negative	0	0	0	0	0	0	0	0	0
XJ06001	1	0	1	1	1	1	1	1	1
XJ06002	1	1	1	1	1	1	1	1	1
XJ06003	0	0	0	0	0	0	0	0	0
XJ06004	0	0	0	0	0	0	0	0	0
XJ06005	0	0	0	0	0	0	0	0	0
XJ06006	0	0	0	0	0	0	0	0	0
XJ06007	0	0	0	0	0	0	0	0	0
XJ06008	0	0	0	0	0	0	0	0	0
XJ06009	0	0	0	0	0	0	0	0	0
XJ06010	0	0	0	0	0	0	0	0	0
XJ06011	0	0	0	0	0	0	0	0	0
XJ06012	0	0	0	0	0	0	0	0	0
XJ06013	1	1	1	1	1	1	1	1	1
XJ06014	1	1	1	0	0	0	0	1	1
XJ06015	0	0	0	0	0	0	0	0	0
XJ06016	0	0	0	0	0	0	0	0	0
XJ06017	1	0	1	1	1	1	1	1	1
XJ06018	1	1	1	0	0	0	0	1	1
XJ06020	0	0	0	0	0	0	0	0	0
XJ06021	1	1	1	1	1	1	1	1	1
XJ06022	0	0	0	0	0	0	0	0	0
XJ06023	0	0	0	0	0	0	0	0	0
XJ06024	0	0	0	0	0	0	0	0	0
XJ06025	1	1	1	1	1	1	1	1	1
XJ06027	0	0	0	0	0	0	0	0	0
XJ06028	0	0	0	0	0	0	0	0	0
XJ06029	0	0	0	0	0	0	0	0	0
XJ06030	0	0	0	0	0	0	0	0	0
XJ06031	0	0	0	0	0	0	0	0	0
XJ06033	1	1	1	0	0	0	0	1	1
XJ06034	1	0	1	1	1	1	1	1	1
XJ06035	0	0	0	0	0	0	0	0	0
XJ06036	1	1	1	1	1	1	1	1	1
XJ06037	0	0	0	0	0	0	0	0	0
XJ06038	1	1	1	1	1	1	1	1	1
XJ06039	1	0	1	1	1	1	1	1	1
XJ06040	1	1	1	0	0	0	0	1	1
XJ06041	0	0	0	0	0	0	0	0	0
XJ06042	1	1	1	1	1	1	1	1	1
XJ06043	0	0	0	0	0	0	0	0	0
XJ06044	0	0	0	0	0	0	0	0	0
XJ06047	1	1	1	1	1	1	1	1	1
XJ06049	0	0	0	0	0	0	0	0	0

XJ06050	0	0	0	0	0	0	0	0	0
XJ06051	1	1	1	1	1	1	1	1	1
XJ06052	1	1	1	1	1	1	1	1	1
XJ06053	0	0	0	0	0	0	0	0	0
XJ06054	0	0	0	0	0	0	0	0	0
XJ06055	1	0	1	0	1	1	1	1	1
XJ06056	1	0	1	1	1	1	1	1	1
XJ06058	0	0	0	0	0	0	0	0	0
XJ06059	1	1	1	1	1	0	1	1	1
XJ06060	1	0	1	1	1	1	1	1	1
XJ06061	0	0	0	0	0	0	0	0	0
XJ06062	0	0	0	0	0	0	0	0	0
XJ06063	1	0	1	1	1	1	1	1	1
XJ06064	0	0	0	0	0	0	0	0	0
XJ06065	0	0	0	0	0	0	0	0	0
XJ06066	0	0	0	0	0	0	0	0	0
XJ06067	0	0	0	0	0	0	0	0	0
XJ06068	0	0	0	0	0	0	0	0	0
XJ06069	0	0	0	0	0	0	0	0	0
XJ06070	0	0	0	0	0	0	0	0	0
XJ06073	0	0	0	0	0	0	0	0	0
XJ06074	0	0	0	0	0	0	0	0	0
XJ06075	1	1	1	0	1	1	0	0	1
XJ06076	0	0	0	0	0	0	0	0	0
XJ06077	0	0	0	0	0	0	0	0	0
XJ06078	0	0	0	0	0	0	0	0	0
XJ06079	0	0	0	0	0	0	0	0	0
XJ06082	0	0	0	0	0	0	0	0	0
XJ06083	1	1	1	1	1	1	1	1	1
XJ06084	0	0	0	0	0	0	0	0	0
XJ06085	0	0	0	0	0	0	0	0	0
XJ06086	0	0	0	0	0	0	0	0	0
XJ06087	0	0	0	0	0	0	0	0	0
XJ06088	0	0	0	0	0	0	0	0	0
XJ06089	1	0	1	1	1	1	1	1	1
XJ06090	1	0	1	1	1	1	1	1	1
XJ06091	0	0	0	0	0	0	0	0	0
XJ06092	0	0	0	0	0	0	0	0	0
XJ06094	0	0	0	0	0	0	0	0	0
XJ06096	0	0	0	0	0	0	0	0	0
XJ06097	0	0	0	0	0	0	0	0	0
XJ06098	1	1	1	1	1	1	1	1	1
XJ06099	0	0	0	0	0	0	0	0	0
XJ06100	0	0	0	0	0	0	0	0	0
XJ06102	0	0	0	0	0	0	0	0	0
XJ06103	0	0	0	0	0	0	0	0	0
XJ06105	0	0	0	0	0	0	0	0	0

XJ06107	0	0	0	0	0	0	0	0	0
XJ06108	1	0	1	1	1	1	1	1	1
XJ06111	0	0	0	0	0	0	0	0	0
XJ06112	0	0	0	0	0	0	0	0	0
XJ06113	0	0	0	0	0	0	0	0	0
XJ06116	1	1	1	1	1	1	1	1	1
XJ06117	0	0	0	0	0	0	0	0	0
XJ06118	0	0	0	0	0	0	0	0	0
XJ06119	0	0	0	0	0	0	0	0	0
XJ06120	0	0	0	0	0	0	0	0	0
XJ06121	0	0	0	0	0	0	0	0	0
XJ06122	0	0	0	0	0	0	0	0	0
XJ06123	0	0	0	0	0	0	0	0	0
XJ06124	0	0	0	0	0	0	0	0	0
XJ06126	0	0	0	0	0	0	0	0	0
XJ06128	0	0	0	0	0	0	0	0	0
XJ06129	0	1	1	0	0	0	0	1	1
XJ06131	0	0	0	0	0	0	0	0	0
XJ06132	1	1	1	1	1	1	1	0	1
XJ06137	0	0	0	0	0	0	0	0	0
XJ06138	0	0	0	0	0	0	0	0	0
XJ06139	0	0	0	0	0	0	0	0	0
XJ06140	0	0	0	0	0	0	0	0	0
XJ06142	1	1	1	1	1	1	0	0	1
XJ06143	0	0	0	0	0	0	0	0	0
XJ06144	0	0	0	0	0	0	0	0	0
XJ06146	0	0	0	0	0	0	0	0	0
XJ06147	0	0	0	0	0	0	0	0	0
XJ06148	0	0	0	0	0	0	0	0	0
XJ06149	1	1	1	1	1	1	0	0	1
XJ06150	1	1	1	1	1	1	0	0	1
XJ06151	0	0	0	0	0	0	0	0	0
XJ06152	0	0	0	0	0	0	0	0	0
XJ06153	1	1	1	0	0	0	0	0	1
XJ06154	0	0	0	0	0	0	0	0	0
XJ06155	1	0	1	0	1	1	0	0	1
XJ06156	1	1	1	1	1	1	1	1	1
XJ06157	0	0	0	0	0	0	0	0	0
XJ06158	1	1	1	0	0	0	0	0	1
XJ06159	1	1	1	0	0	0	0	0	1
XJ06160	1	1	1	1	1	0	0	0	0
XJ06161	1	1	0	1	1	1	1	1	1
XJ06162	0	0	0	0	0	0	0	0	0
XJ06164	1	1	1	1	1	1	1	1	1
XJ06165	0	0	0	0	0	0	0	0	0
XJ06166	0	0	0	0	0	0	0	0	0
XJ06167	0	0	0	0	0	0	0	0	0

XJ06168	0	0	0	0	0	0	0	0	0
XJ06169	0	0	0	0	0	0	0	0	0
XJ06170	0	0	0	0	0	0	0	0	0
XJ06171	1	1	1	1	1	1	1	1	1
XJ06172	0	0	0	0	0	0	0	0	0
XJ06173	0	0	0	0	0	0	0	0	0
XJ06174	0	0	0	0	0	0	0	0	0
XJ06175	0	0	0	0	0	0	0	0	0
XJ06176	1	1	1	1	1	1	1	1	1
XJ06177	1	1	1	1	1	1	1	1	1
XJ06178	0	0	0	0	0	0	0	0	0
XJ06179	0	0	0	0	0	0	0	0	0
XJ06180	0	0	0	0	0	0	0	0	0
XJ06181	0	0	0	0	0	0	0	0	0
XJ06182	1	1	1	1	1	1	1	1	1
XJ06183	1	1	1	1	1	1	1	1	1
XJ06184	0	0	0	0	0	0	0	0	0
XJ06185	0	0	0	0	0	0	0	0	0
XJ06186	1	0	1	1	1	1	1	1	1
XJ06187	0	0	0	0	0	0	0	0	0
XJ06188	1	1	1	0	0	0	0	1	1
XJ06189	0	0	0	0	0	0	0	0	0
XJ06191	0	0	0	0	0	0	0	0	0
XJ06192	1	1	1	1	1	1	1	1	1
XJ06193	0	0	0	0	0	0	0	0	0
XJ06194	0	0	0	0	0	0	0	0	0
XJ06195	1	1	1	1	1	1	1	1	1
XJ06196	1	1	1	1	1	1	1	1	1
XJ06197	1	1	1	1	1	1	1	1	1
XJ06201	0	0	0	0	0	0	0	0	0
XJ06202	0	0	0	0	0	0	0	0	0
XJ06203	0	0	0	0	0	0	0	0	0
XJ06204	0	0	0	0	0	0	0	0	0
XJ06205	0	0	0	0	0	0	0	0	0
XJ11003	0	0	0	0	0	0	0	0	0
XJ11007	0	0	0	0	0	0	0	0	0
XJ11008	1	1	1	1	1	1	1	1	1
XJ11009	1	0	1	1	1	1	1	1	1
XJ11010	0	0	0	0	0	0	0	0	0
XJ11013	1	1	1	1	1	1	1	1	1
XJ11014	0	0	0	0	0	0	0	0	0
XJ11015	0	0	0	0	0	0	0	0	0
XJ11016	0	0	0	0	0	0	0	0	0
XJ11017	1	1	1	1	1	1	1	1	1
XJ11019	0	0	0	0	0	0	0	0	0
XJ11020	1	0	1	1	1	1	1	1	1
XJ11021	0	0	0	0	0	0	0	0	0
XJ11022	1	1	1	1	1	1	1	1	1

XJ11023	0	0	0	0	0	0	0	0	0
XJ11024	0	0	0	0	0	0	0	0	0
XJ11025	0	0	0	0	0	0	0	0	0
XJ11026	1	1	1	1	1	1	1	1	1
XJ11027	0	0	0	0	0	0	0	0	0
XJ11028	0	0	0	0	0	0	0	0	0
XJ11029	0	0	0	0	0	0	0	0	0
XJ11030	0	0	0	0	0	0	0	0	0
XJ11031	0	0	0	0	0	0	0	0	0
XJ11032	0	0	0	0	0	0	0	0	0
XJ11033	0	0	0	0	0	0	0	0	0
XJ11034	0	0	0	0	0	0	0	0	0
XJ11035	0	0	0	0	0	0	0	0	0
XJ11036	0	0	0	0	0	0	0	0	0
XJ11037	0	0	0	0	0	0	0	0	0
XJ11038	0	0	0	0	0	0	0	0	0
XJ11039	0	0	0	0	0	0	0	0	0
XJ11042	0	0	0	0	0	0	0	0	0
XJ11043	0	0	0	0	0	0	0	0	0
XJ11044	0	0	0	0	0	0	0	0	0
XJ11045	0	0	0	0	0	0	0	0	0
XJ11047	1	1	1	1	1	1	1	1	1
XJ11048	0	0	0	0	0	0	0	0	0
XJ11049	1	1	1	0	0	0	0	1	1
XJ11050	0	0	0	0	0	0	0	0	0
XJ11051	1	0	1	1	1	1	1	1	1
XJ11052	0	0	0	0	0	0	0	0	0
XJ11053	0	0	0	0	0	0	0	0	0
XJ11054	0	0	0	0	0	0	0	0	0
XJ11055	0	0	0	0	0	0	0	0	0
XJ11056	1	1	1	0	0	0	0	1	1
XJ11058	1	1	1	1	1	1	1	1	1
XJ11059	0	0	0	0	0	0	0	0	0
XJ11060	0	0	0	0	0	0	0	0	0
XJ11061	0	0	0	0	0	0	0	0	0
XJ11062	1	0	1	1	0	1	1	1	1
XJ11063	0	0	0	0	0	0	0	0	0
XJ11064	1	1	1	1	1	1	1	1	1
XJ11065	0	0	0	0	0	0	0	0	0
XJ11066	0	0	0	0	0	0	0	0	0
XJ11067	0	0	0	0	0	0	0	0	0
XJ11070	0	0	0	0	0	0	0	0	0
XJ11071	1	1	0	1	1	1	1	1	1
XJ11072	0	0	0	0	0	0	0	0	0
XJ11073	1	1	1	1	0	1	1	1	1
XJ11074	0	0	0	0	0	0	0	0	0
XJ11075	0	0	0	0	0	0	0	0	0
XJ11076	0	0	0	0	0	0	0	0	0
XJ11077	0	0	0	0	0	0	0	0	0
XJ11078	1	1	1	1	0	1	1	1	1

XJ11079	0	0	0	0	0	0	0	0	0
XJ11080	0	0	0	0	0	0	0	0	0
XJ11081	0	0	0	0	0	0	0	0	0
XJ11082	0	0	0	0	0	0	0	0	0
XJ11083	0	0	0	0	0	0	0	0	0
XJ11084	0	0	0	0	0	0	0	0	0
XJ11085	0	0	0	0	0	0	0	0	0
XJ11086	0	0	0	0	0	0	0	0	0
XJ11087	1	1	1	1	1	1	1	1	1
XJ11088	0	0	0	0	0	0	0	0	0
XJ11089	0	0	0	0	0	0	0	0	0
XJ11090	1	1	1	1	1	1	1	1	1
XJ11091	0	0	0	0	0	0	0	0	0
XJ11092	0	0	0	0	0	0	0	0	0
XJ11094	0	0	0	0	0	0	0	0	0
XJ11095	0	0	0	0	0	0	0	0	0
XJ11096	0	0	0	0	0	0	0	0	0
XJ11097	1	1	1	1	1	1	1	1	1
XJ11098	0	0	0	0	0	0	0	0	0
XJ11099	0	0	0	0	0	0	0	0	0
XJ11100	0	0	0	0	0	0	0	0	0
XJ11101	0	0	0	0	0	0	0	0	0
XJ11102	0	0	0	0	0	0	0	0	0
XJ11103	0	0	0	0	0	0	0	0	0
XJ11104	0	0	0	0	0	0	0	0	0
XJ11105	1	1	1	0	0	0	0	1	1
XJ11106	0	0	0	0	0	0	0	0	0
XJ11107	1	1	1	1	1	1	1	1	1
XJ11108	0	0	0	0	0	0	0	0	0
XJ11109	0	0	0	0	0	0	0	0	0
XJ11110	0	0	0	0	0	0	0	0	0
XJ11111	0	0	0	0	0	0	0	0	0
XJ11113	0	0	0	0	0	0	0	0	0
XJ11114	0	0	0	0	0	0	0	0	0
XJ11115	0	0	0	0	0	0	0	0	0
XJ11116	0	0	0	0	0	0	0	0	0
XJ11117	0	0	0	0	0	0	0	0	0
XJ11118	1	1	1	0	0	0	0	1	1
XJ11119	0	0	0	0	0	0	0	0	0
XJ11120	0	0	0	0	0	0	0	0	0
XJ11121	0	0	0	0	0	0	0	0	0
XJ11122	0	0	0	0	0	0	0	0	0
XJ11123	0	0	0	0	0	0	0	0	0
XJ11125	0	0	0	0	0	0	0	0	0
XJ11126	0	0	0	0	0	0	0	0	0
XJ11127	0	0	0	0	0	0	0	0	0
XJ11128	0	0	0	0	0	0	0	0	0
XJ11130	0	0	0	0	0	0	0	0	0
XJ11131	1	1	1	1	1	1	1	1	1
XJ11132	1	0	1	0	0	0	0	1	1

XJ11133	0	0	0	0	0	0	0	0	0
XJ11134	0	0	0	0	0	0	0	0	0
XJ11135	0	0	0	0	0	0	0	0	0
XJ11137	1	1	1	0	0	0	0	1	1
XJ11138	1	1	1	1	0	0	0	0	0
XJ11139	0	0	0	0	0	0	0	0	0
XJ11140	1	1	1	1	1	1	1	1	1
XJ11141	0	0	0	0	0	0	0	0	0
XJ11142	0	0	0	0	0	0	0	0	0
XJ11143	0	0	0	0	0	0	0	0	0
XJ11144	0	0	0	0	0	0	0	0	0
XJ11145	1	1	1	0	0	0	0	1	1
XJ11146	0	0	0	0	0	0	0	0	0
XJ11147	0	0	0	0	0	0	0	0	0
XJ11148	0	0	0	0	0	0	0	0	0
XJ11149	0	0	0	0	0	0	0	0	0
XJ11151	1	1	1	0	0	0	0	1	1
XJ11152	1	1	1	1	1	1	1	1	1
XJ11153	0	0	0	0	0	0	0	0	0
XJ11154	0	0	0	0	0	0	0	0	0
XJ11156	1	1	1	0	0	0	0	1	1
XJ11157	1	0	1	1	1	1	0	0	1
XJ11158	0	0	0	0	0	0	0	0	0
XJ11159	0	0	0	0	0	0	0	0	0
XJ11160	0	0	0	0	0	0	0	0	0
XJ11161	0	0	0	0	0	0	0	0	0

10	11	12	13	14	15	16	17	18	19
1	1	1	1	1	1	1	1	1	1
1	1	1	1	1	1	0	1	1	1
0	0	0	0	0	0	0	0	0	0
1	1	1	1	0	1	1	1	0	0
1	1	1	1	1	1	1	1	1	1
0	0	0	0	0	0	0	0	0	0
0	0	0	0	0	0	0	0	0	0
0	0	0	0	0	0	0	0	0	0
0	0	0	0	0	0	0	0	0	0
0	0	0	0	0	0	0	0	0	0
0	0	0	0	0	0	0	0	0	0
0	0	0	0	0	0	0	0	0	0
0	0	0	0	0	0	0	0	0	0
0	0	0	0	0	0	0	0	0	0
0	0	0	0	0	0	0	0	0	0
1	1	1	1	1	1	1	1	1	1
0	1	1	1	1	1	1	1	1	1
0	0	0	0	0	0	0	0	0	0
0	0	0	0	0	0	0	0	0	0
1	1	1	1	1	1	1	1	1	1
1	1	1	1	1	1	1	1	1	1
0	0	0	0	0	0	0	0	0	0
1	1	1	1	1	1	1	1	1	1
0	0	0	0	0	0	0	0	0	0
0	0	0	0	0	0	0	0	0	0
0	0	0	0	0	0	0	0	0	0
1	1	1	1	1	1	1	1	1	1
0	0	0	0	0	0	0	0	0	0
0	0	0	0	0	0	0	0	0	0
0	0	0	0	0	0	0	0	0	0
0	0	0	0	0	0	0	0	0	0
0	0	0	0	0	0	0	0	0	0
1	1	1	1	1	1	1	1	1	1
1	1	1	1	1	1	1	1	1	1
0	0	0	0	0	0	0	0	0	0
1	1	1	1	0	0	0	0	0	0
0	0	0	0	0	0	0	0	0	0
1	1	1	1	0	0	0	0	0	0
1	1	1	1	1	1	1	1	1	1
1	1	1	1	1	1	1	1	1	1
0	0	0	0	0	0	0	0	0	0
1	1	1	0	1	1	1	1	1	1
0	0	0	0	0	0	0	0	0	0
0	0	0	0	0	0	0	0	0	0
1	1	1	1	1	1	1	1	1	1
0	0	0	0	0	0	0	0	0	0

0	0	0	0	0	0	0	0	0	0
1	1	1	1	0	0	1	1	1	1
1	1	1	1	1	1	1	1	1	1
0	0	0	0	0	0	0	0	0	0
0	0	0	0	0	0	0	0	0	0
1	1	1	1	1	1	1	1	1	1
1	1	1	1	1	1	1	1	1	1
0	0	0	0	0	0	0	0	0	0
1	1	1	1	0	1	1	1	1	1
1	1	1	1	1	1	1	1	1	1
0	0	0	0	0	0	0	0	0	0
0	0	0	0	0	0	0	0	0	0
1	1	1	1	1	1	1	1	1	1
0	0	0	0	0	0	0	0	0	0
0	0	0	0	0	0	0	0	0	0
0	0	0	0	0	0	0	0	0	0
0	0	0	0	0	0	0	0	0	0
0	0	0	0	0	0	0	0	0	0
0	0	0	0	0	0	0	0	0	0
0	0	0	0	0	0	0	0	0	0
0	0	0	0	0	0	0	0	0	0
0	0	0	0	0	0	0	0	0	0
1	1	1	0	1	1	1	1	1	1
0	0	0	0	0	0	0	0	0	0
0	0	0	0	0	0	0	0	0	0
0	0	0	0	0	0	0	0	0	0
0	0	0	0	0	0	0	0	0	0
0	0	0	0	0	0	0	0	0	0
1	1	1	1	0	0	0	0	1	0
0	0	0	0	0	0	0	0	0	0
0	0	0	0	0	0	0	0	0	0
0	0	0	0	0	0	0	0	0	0
0	0	0	0	0	0	0	0	0	0
0	0	0	0	0	0	0	0	0	0
1	1	1	1	1	1	1	1	1	1
1	1	1	1	1	1	1	1	1	1
0	0	0	0	0	0	0	0	0	0
0	0	0	0	0	0	0	0	0	0
0	0	0	0	0	0	0	0	0	0
0	0	0	0	0	0	0	0	0	0
0	0	0	0	0	0	0	0	0	0
1	1	1	1	1	1	1	1	1	1
0	0	0	0	0	0	0	0	0	0
0	0	0	0	0	0	0	0	0	0
0	0	0	0	0	0	0	0	0	0
0	0	0	0	0	0	0	0	0	0
0	0	0	0	0	0	0	0	0	0

0	0	0	0	0	0	0	0	0	0
1	1	1	0	0	1	1	1	1	1
0	0	0	0	0	0	0	0	0	0
0	0	0	0	0	0	0	0	0	0
0	0	0	0	0	0	0	0	0	0
1	1	1	0	1	1	1	1	1	1
0	0	0	0	0	0	0	0	0	0
0	0	0	0	0	0	0	0	0	0
0	0	0	0	0	0	0	0	0	0
0	0	0	0	0	0	0	0	0	0
0	0	0	0	0	0	0	0	0	0
0	0	0	0	0	0	0	0	0	0
0	0	0	0	0	0	0	0	0	0
0	0	0	0	0	0	0	0	0	0
0	0	0	0	0	0	0	0	0	0
0	0	0	0	0	0	0	0	0	0
1	1	1	1	1	1	1	1	1	1
0	0	0	0	0	0	0	0	0	0
1	1	1	1	1	1	1	1	1	1
0	0	0	0	0	0	0	0	0	0
0	0	0	0	0	0	0	0	0	0
0	0	0	0	0	0	0	0	0	0
0	0	0	0	0	0	0	0	0	0
1	1	1	1	1	1	1	1	1	1
0	0	0	0	0	0	0	0	0	0
0	0	0	0	0	0	0	0	0	0
0	0	0	0	0	0	0	0	0	0
0	0	0	0	0	0	0	0	0	0
0	0	0	0	0	0	0	0	0	0
1	1	1	1	0	0	1	1	1	1
1	1	1	1	1	1	1	1	1	1
0	0	0	0	0	0	0	0	0	0
0	0	0	0	0	0	0	0	0	0
1	1	1	1	1	1	1	1	1	1
0	0	0	0	0	0	0	0	0	0
1	1	1	1	1	0	0	0	1	1
1	1	1	1	1	1	1	1	1	1
0	0	0	0	0	0	0	0	0	0
1	1	1	1	1	1	1	1	1	1
1	1	1	1	1	1	1	1	1	1
1	1	1	1	1	1	1	1	1	1
1	0	0	1	1	1	1	1	1	1
0	0	0	0	0	0	0	0	0	0
1	1	1	1	0	0	0	1	1	1
0	0	0	0	0	0	0	0	0	0
0	0	0	0	0	0	0	0	0	0
0	0	0	0	0	0	0	0	0	0

0	0	0	0	0	0	0	0	0	0
0	0	0	0	0	0	0	0	0	0
0	0	0	0	0	0	0	0	0	0
1	1	1	1	0	0	1	1	1	1
0	0	0	0	0	0	0	0	0	0
0	0	0	0	0	0	0	0	0	0
0	0	0	0	0	0	0	0	0	0
0	0	0	0	0	0	0	0	0	0
1	1	1	1	1	1	1	1	1	1
1	1	1	1	1	1	1	1	1	1
0	0	0	0	0	0	0	0	0	0
0	0	0	0	0	0	0	0	0	0
0	0	0	0	0	0	0	0	0	0
0	0	0	0	0	0	0	0	0	0
1	1	1	1	1	1	1	1	1	1
1	1	1	0	1	1	1	1	1	1
0	0	0	0	0	0	0	0	0	0
0	0	0	0	0	0	0	0	0	0
1	1	1	1	1	1	1	1	1	1
0	0	0	0	0	0	0	0	0	0
1	1	1	1	1	1	1	1	1	1
0	0	0	0	0	0	0	0	0	0
0	0	0	0	0	0	0	0	0	0
1	1	1	1	1	1	1	1	1	1
0	0	0	0	0	0	0	0	0	0
0	0	0	0	0	0	0	0	0	0
1	1	1	1	0	0	0	0	0	0
1	1	1	1	1	1	1	1	1	1
1	1	1	1	1	1	1	1	1	1
0	0	0	0	0	0	0	0	0	0
0	0	0	0	0	0	0	0	0	0
0	0	0	0	0	0	0	0	0	0
0	0	0	0	0	0	0	0	0	0
0	0	0	0	0	0	0	0	0	0
0	0	0	0	0	0	0	0	0	0
0	0	0	0	0	0	0	0	0	0
1	1	1	1	1	0	1	1	1	1
1	1	1	1	1	1	1	1	1	1
0	0	0	0	0	0	0	0	0	0
1	1	1	1	1	1	1	1	1	1
0	0	0	0	0	0	0	0	0	0
0	0	0	0	0	0	0	0	0	0
0	0	0	0	0	0	0	0	0	0
1	1	1	1	1	1	1	1	1	1
0	0	0	0	0	0	0	0	0	0
1	1	1	1	1	1	1	1	1	1
0	0	0	0	0	0	0	0	0	0
1	1	1	1	1	1	1	1	1	1

0	0	0	0	0	0	0	0	0	0
0	0	0	0	0	0	0	0	0	0
0	0	0	0	0	0	0	0	0	0
1	1	1	1	1	1	1	1	1	1
0	0	0	0	0	0	0	0	0	0
0	0	0	0	0	0	0	0	0	0
0	0	0	0	0	0	0	0	0	0
0	0	0	0	0	0	0	0	0	0
0	0	0	0	0	0	0	0	0	0
0	0	0	0	0	0	0	0	0	0
0	0	0	0	0	0	0	0	0	0
0	0	0	0	0	0	0	0	0	0
0	0	0	0	0	0	0	0	0	0
0	0	0	0	0	0	0	0	0	0
0	0	0	0	0	0	0	0	0	0
0	0	0	0	0	0	0	0	0	0
0	0	0	0	0	0	0	0	0	0
0	0	0	0	0	0	0	0	0	0
0	0	0	0	0	0	0	0	0	0
0	0	0	0	0	0	0	0	0	0
0	0	0	0	0	0	0	0	0	0
1	1	1	1	1	1	1	1	1	1
0	0	0	0	0	0	0	0	0	0
1	1	0	0	0	0	0	1	1	1
0	0	0	0	0	0	0	0	0	0
1	1	1	1	1	1	1	1	1	1
0	0	0	0	0	0	0	0	0	0
0	0	0	0	0	0	0	0	0	0
0	0	0	0	0	0	0	0	0	0
0	0	0	0	0	0	0	0	0	0
1	1	1	1	1	1	1	1	1	1
1	1	1	1	1	1	1	1	1	1
0	0	0	0	0	0	0	0	0	0
0	0	0	0	0	0	0	0	0	0
0	0	0	0	0	0	0	0	0	0
1	1	1	0	0	1	1	1	1	1
0	0	0	0	0	0	0	0	0	0
1	1	1	1	1	1	1	1	1	1
0	0	0	0	0	0	0	0	0	0
0	0	0	0	0	0	0	0	0	0
0	0	0	0	0	0	0	0	0	0
0	0	0	0	0	0	0	0	0	0
1	1	1	1	1	1	1	1	1	1
0	0	0	0	0	0	0	0	0	0
1	1	1	1	1		1	1	1	1
0	0	0	0	0	0	0	0	0	0
0	0	0	0	0	0	0	0	0	0
0	0	0	0	0	0	0	0	0	0
0	0	0	0	0	0	0	0	0	0
1	1	1	1	1	1	1	1	1	1

0	0	0	0	0	0	0	0	0	0
0	0	0	0	0	0	0	0	0	0
0	0	0	0	0	0	0	0	0	0
0	0	0	0	0	0	0	0	0	0
0	0	0	0	0	0	0	0	0	0
0	0	0	0	0	0	0	0	0	0
0	0	0	0	0	0	0	0	0	0
0	0	0	0	0	0	0	0	0	0
1	1	1	0	1	1	1	1	1	1
0	0	0	0	0	0	0	0	0	0
0	0	0	0	0	0	0	0	0	0
1	1	1	1	1	1	1	1	1	1
0	0	0	0	0	0	0	0	0	0
0	0	0	0	0	0	0	0	0	0
0	0	0	0	0	0	0	0	0	0
0	0	0	0	0	0	0	0	0	0
0	0	0	0	0	0	0	0	0	0
0	1	1	1	1	1	1	1	1	1
0	0	0	0	0	0	0	0	0	0
0	0	0	0	0	0	0	0	0	0
0	0	0	0	0	0	0	0	0	0
0	0	0	0	0	0	0	0	0	0
0	0	0	0	0	0	0	0	0	0
0	0	0	0	0	0	0	0	0	0
0	0	0	0	0	0	0	0	0	0
1	1	1	1	1	1	1	1	1	1
0	0	0	0	0	0	0	0	0	0
0	0	0	0	1	1	1	1	1	
0	0	0	0	0	0	0	0	0	0
0	0	0	0	0	0	0	0	0	0
0	0	0	0	0	0	0	0	0	0
0	0	0	0	0	0	0	0	0	0
0	0	0	0	0	0	0	0	0	0
0	0	0	0	0	0	0	0	0	0
0	0	0	0	0	0	0	0	0	0
0	0	0	0	0	0	0	0	0	0
0	0	0	0	0	0	0	0	0	0
1	1	1	1	1	1	1	1	1	1
0	0	0	0	0	0	0	0	0	0
0	0	0	0	0	0	0	0	0	0
0	0	0	0	0	0	0	0	0	0
0	0	0	0	0	0	0	0	0	0
0	0	0	0	0	0	0	0	0	0
0	0	0	0	0	0	0	0	0	0
0	0	0	0	0	0	0	0	0	0
0	0	0	0	0	0	0	0	0	0
0	0	0	0	0	0	0	0	0	0
0	0	0	0	0	0	0	0	0	0
1	1	1	1	1	1	1	1	1	1
1	1	1	1	1	1	1	1	1	1

0	0	0	0	0	0	0	0	0	0
0	0	0	0	0	0	0	0	0	0
0	0	0	0	0	0	0	0	0	0
1	1	1	1	1	1	1	1	1	1
1	1	0	0	0	1	1	1	1	1
0	0	0	0	0	0	0	0	0	0
1	1	1	1	1	1	1	1	1	1
0	0	0	0	0	0	0	0	0	0
0	0	0	0	0	0	0	0	0	0
0	0	0	0	0	0	0	0	0	0
0	0	0	0	0	0	0	0	0	0
1	1	1	1	1	1	1	1	1	1
0	0	0	0	0	0	0	0	0	0
0	0	0	0	0	0	0	0	0	0
0	0	0	0	0	0	0	0	0	0
0	0	0	0	0	0	0	0	0	0
1	1	1	1	1	1	1	1	1	1
1	1	1	1	1	0	1	1	1	1
0	0	0	0	0	0	0	0	0	0
0	0	0	0	0	0	0	0	0	0
1	1	1	1	1	1	1	1	0	0
0	0	0	0	0	0	0	0	0	0
0	0	0	0	0	0	0	0	0	0
0	0	0	0	0	0	0	0	0	0
0	0	0	0	0	0	0	0	0	0
0	0	0	0	0	0	0	0	0	0

20	21	22	23	24	25	26	27	28	29
0	0	1	1	1	1	1	1	1	1
1	1	1	1	1	1	1	1	1	1
0	0	0	0	0	0	0	0	0	0
0	0	0	0	0	0	0	1	1	1
1	1	1	1	1	1	1	1	1	1
0	0	0	0	0	0	0	0	0	0
0	0	0	0	0	0	0	0	0	0
0	0	0	0	0	0	0	0	0	0
0	0	0	0	0	0	0	0	0	0
0	0	0	0	0	0	0	0	0	0
0	0	0	0	0	0	0	0	0	0
0	0	0	0	0	0	0	0	0	0
0	0	0	0	0	0	0	0	0	0
0	0	0	0	0	0	0	0	0	0
0	0	0	0	0	0	0	0	0	0
1	1	1	1	1	1	1	1	1	1
1	1	1	0	0	0	0	0	0	0
0	0	0	0	0	0	0	0	0	0
0	0	0	0	0	0	0	0	0	0
1	0	1	1	1	1	1	1	1	1
1	1	1	0	0	0	0	0	0	0
0	0	0	0	0	0	0	0	0	0
1	1	1	1	1	1	1	1	1	1
0	0	0	0	0	0	0	0	0	0
0	0	0	0	0	0	0	0	0	0
0	0	0	0	0	0	0	0	0	0
1	1	1	1	1	1	1	1	1	1
0	0	0	0	0	0	0	0	0	0
0	0	0	0	0	0	0	0	0	0
0	0	0	0	0	0	0	0	0	0
0	0	0	0	0	0	0	0	0	0
0	0	0	0	0	0	0	0	0	0
1	1	1	0	0	0	0	0	0	0
1	1	1	1	1	1	1	1	1	1
0	0	0	0	0	0	0	0	0	0
0	0	0	0	0	0	0	0	0	0
0	0	0	0	0	0	0	0	0	0
0	0	0	0	0	1	1	1	1	1
1	1	1	1	1	1	1	1	1	0
1	1	1	0	0	1	1	1	1	1
0	0	0	0	0	0	0	0	0	0
1	1	1	1	1	1	1	1	1	0
0	0	0	0	0	0	0	0	0	0
0	0	0	0	0	0	0	0	0	0
1	1	1	1	1	1	1	1	1	1
0	0	0	0	0	0	0	0	0	0

0	0	0	0	0	0	0	0	0	0
1	1	1	1	1	1	1	1	1	1
1	1	1	1	1	1	1	1	1	1
0	0	0	0	0	0	0	0	0	0
0	0	0	0	0	0	0	0	0	0
1	1	1	1	1	1	1	1	1	0
1	1	0	1	1	1	1	1	1	0
0	0	0	0	0	0	0	0	0	0
1	1	1	1	1	1	1	1	1	1
1	1	1	1	1	1	1	1	1	0
0	0	0	0	0	0	0	0	0	0
0	0	0	0	0	0	0	0	0	0
1	1	1	1	1	1	1	1	1	0
0	0	0	0	0	0	0	0	0	0
0	0	0	0	0	0	0	0	0	0
0	0	0	0	0	0	0	0	0	0
0	0	0	0	0	0	0	0	0	0
0	0	0	0	0	0	0	0	0	0
0	0	0	0	0	0	0	0	0	0
0	0	0	0	0	0	0	0	0	0
0	0	0	0	0	0	0	0	0	0
0	0	0	0	0	0	0	0	0	0
1	1	1	1	1	1	1	1	1	0
0	0	0	0	0	0	0	0	0	0
0	0	0	0	0	0	0	0	0	0
0	0	0	0	0	0	0	0	0	0
0	0	0	0	0	0	0	0	0	0
0	0	0	0	0	0	0	0	0	0
0	0	0	0	0	0	1	1	1	1
0	0	0	0	0	0	0	0	0	0
0	0	0	0	0	0	0	0	0	0
0	0	0	0	0	0	0	0	0	0
0	0	0	0	0	0	0	0	0	0
0	0	0	0	0	0	0	0	0	0
1	1	1	1	1	1	1	1	1	0
1	1	1	1	1	1	1	1	1	0
0	0	0	0	0	0	0	0	0	0
0	0	0	0	0	0	0	0	0	0
0	0	0	0	0	0	0	0	0	0
0	0	0	0	0	0	0	0	0	0
0	0	0	0	0	0	0	0	0	0
1	1	0	1	1	1	1	1	1	1
0	0	0	0	0	0	0	0	0	0
0	0	0	0	0	0	0	0	0	0
0	0	0	0	0	0	0	0	0	0
0	0	0	0	0	0	0	0	0	0
0	0	0	0	0	0	0	0	0	0

0	0	0	0	0	0	0	0	0	0
1	1	1	1	1	1	1	1	1	0
0	0	0	0	0	0	0	0	0	0
0	0	0	0	0	0	0	0	0	0
0	0	0	0	0	0	0	0	0	0
1	1	1	1	1	0	0	0	0	0
0	0	0	0	0	0	0	0	0	0
0	0	0	0	0	0	0	0	0	0
0	0	0	0	0	0	0	0	0	0
0	0	0	0	0	0	0	0	0	0
0	0	0	0	0	0	0	0	0	0
0	0	0	0	0	0	0	0	0	0
0	0	0	0	0	0	0	0	0	0
0	0	0	0	0	0	0	0	0	0
0	0	0	0	0	0	0	0	0	0
0	0	0	0	0	0	0	0	0	0
1	1	1	0	0	0	0	0	0	0
0	0	0	0	0	0	0	0	0	0
1	0	1	1	1	1	1	1	1	1
0	0	0	0	0	0	0	0	0	0
0	0	0	0	0	0	0	0	0	0
0	0	0	0	0	0	0	0	0	0
0	0	0	0	0	0	0	0	0	0
1	1	1	1	1	1	1	1	1	1
0	0	0	0	0	0	0	0	0	0
0	0	0	0	0	0	0	0	0	0
0	0	0	0	0	0	0	0	0	0
0	0	0	0	0	0	0	0	0	0
0	0	0	0	0	0	0	0	0	0
1	0	0	0	1	1	1	1	1	1
1	0	1	1	1	1	1	1	1	1
0	0	0	0	0	0	0	0	0	0
0	0	0	0	0	0	0	0	0	0
1	0	1	0	0	0	0	0	0	0
0	0	0	0	0	0	0	0	0	0
1	1	1	1	1	1	1	1	1	0
1	1	1	1	1	1	1	1	1	1
0	0	0	0	0	0	0	0	0	0
1	1	1	0	0	0	0	0	0	0
1	1	1	0	0	0	0	0	0	0
1	1	1	1	1	1	1	1	1	1
1	1	1	1	1	1	1	1	1	1
0	0	0	0	0	0	0	0	0	0
1	1	1	1	0	1	1	1	1	1
0	0	0	0	0	0	0	0	0	0
0	0	0	0	0	0	0	0	0	0
0	0	0	0	0	0	0	0	0	0

0	0	0	0	0	0	0	0	0	0
0	0	0	0	0	0	0	0	0	0
0	0	0	0	0	0	0	0	0	0
1	1	1	1	1	1	1	1	1	1
0	0	0	0	0	0	0	0	0	0
0	0	0	0	0	0	0	0	0	0
0	0	0	0	0	0	0	0	0	0
0	0	0	0	0	0	0	0	0	0
1	1	1	1	1	1	1	1	1	1
1	1	1	1	1	1	1	1	1	1
0	0	0	0	0	0	0	0	0	0
0	0	0	0	0	0	0	0	0	0
0	0	0	0	0	0	0	0	0	0
0	0	0	0	0	0	0	0	0	0
1	1	1	1	1	1	1	1	1	1
1	1	1	1	1	0	0	0	0	0
0	0	0	0	0	0	0	0	0	0
0	0	0	0	0	0	0	0	0	0
1	1	1	1	1	1	1	1	1	0
0	0	0	0	0	0	0	0	0	0
1	1	1	0	0	0	0	0	0	0
0	0	0	0	0	0	0	0	0	0
0	0	0	0	0	0	0	0	0	0
1	1	1	1	1	1	1	1	1	1
0	0	0	0	0	0	0	0	0	0
0	0	0	0	0	0	0	0	0	0
0	0	0	0	0	0	1	1	1	1
1	1	1	1	1	1	1	1	1	1
1	1	1	1	1	1	1	1	1	1
0	0	0	0	0	0	0	0	0	0
0	0	0	0	0	0	0	0	0	0
0	0	0	0	0	0	0	0	0	0
0	0	0	0	0	0	0	0	0	0
0	0	0	0	0	0	0	0	0	0
0	0	0	0	0	0	0	0	0	0
0	0	0	0	0	0	0	0	0	0
1	1	1	1	1	1	1	1	1	1
1	1	1	1	1	1	1	1	1	0
0	0	0	0	0	0	0	0	0	0
1	1	1	1	1	1	1	1	1	1
0	0	0	0	0	0	0	0	0	0
0	0	0	0	0	0	0	0	0	0
0	0	0	0	0	0	0	0	0	0
1	1	1	1	1	1	1	1	1	1
0	0	0	0	0	0	0	0	0	0
1	1	1	1	1	1	1	1	1	0
0	0	0	0	0	0	0	0	0	0
1	1	1	1	1	1	1	1	1	1

0	0	0	0	0	0	0	0	0	0
0	0	0	0	0	0	0	0	0	0
0	0	0	0	0	0	0	0	0	0
1	1	1	1	1	1	1	1	1	1
0	0	0	0	0	0	0	0	0	0
0	0	0	0	0	0	0	0	0	0
0	0	0	0	0	0	0	0	0	0
0	0	0	0	0	0	0	0	0	0
0	0	0	0	0	0	0	0	0	0
0	0	0	0	0	0	0	0	0	0
0	0	0	0	0	0	0	0	0	0
0	0	0	0	0	0	0	0	0	0
0	0	0	0	0	0	0	0	0	0
0	0	0	0	0	0	0	0	0	0
0	0	0	0	0	0	0	0	0	0
0	0	0	0	0	0	0	0	0	0
0	0	0	0	0	0	0	0	0	0
0	0	0	0	0	0	0	0	0	0
0	0	0	0	0	0	0	0	0	0
0	0	0	0	0	0	0	0	0	0
0	0	0	0	0	0	0	0	0	0
1	1	1	1	1	1	1	1	1	1
0	0	0	0	0	0	0	0	0	0
1	1	1	0	0	0	0	0	0	0
0	0	0	0	0	0	0	0	0	0
1	1	1	1	1	1	1	1	1	0
0	0	0	0	0	0	0	0	0	0
0	0	0	0	0	0	0	0	0	0
0	0	0	0	0	0	0	0	0	0
0	0	0	0	0	0	0	0	0	0
1	1	1	0	0	0	0	0	0	0
1	1	1	1	1	1	1	1	1	1
0	0	0	0	0	0	0	0	0	0
0	0	0	0	0	0	0	0	0	0
0	0	0	0	0	0	0	0	0	0
1	1	1	1	1	1	1	1	1	0
0	0	0	0	0	0	0	0	0	0
1	1	1	1	1	1	1	1	1	1
0	0	0	0	0	0	0	0	0	0
0	0	0	0	0	0	0	0	0	0
0	0	0	0	0	0	0	0	0	0
0	0	0	0	0	0	0	0	0	0
1	1	1	1	1	1	1	1	1	1
0	0	0	0	0	0	0	0	0	0
1	1	1	1	1	1	1	1	1	1
0	0	0	0	0	0	0	0	0	0
0	0	0	0	0	0	0	0	0	0
0	0	0	0	0	0	0	0	0	0
0	0	0	0	0	0	0	0	0	0
1	1	1	1	1	1	1	1	1	1

0	0	0	0	0	0	0	0	0	0
0	0	0	0	0	0	0	0	0	0
0	0	0	0	0	0	0	0	0	0
0	0	0	0	0	0	0	0	0	0
0	0	0	0	0	0	0	0	0	0
0	0	0	0	0	0	0	0	0	0
0	0	0	0	0	0	0	0	0	0
0	0	0	0	0	0	0	0	0	0
1	1	1	1	1	1	1	1	1	0
0	0	0	0	0	0	0	0	0	0
0	0	0	0	0	0	0	0	0	0
1	1	1	1	1	1	1	1	1	1
0	0	0	0	0	0	0	0	0	0
0	0	0	0	0	0	0	0	0	0
0	0	0	0	0	0	0	0	0	0
0	0	0	0	0	0	0	0	0	0
0	0	0	0	0	0	0	0	0	0
1	1	1	1	1	1	1	1	1	1
0	0	0	0	0	0	0	0	0	0
0	0	0	0	0	0	0	0	0	0
0	0	0	0	0	0	0	0	0	0
0	0	0	0	0	0	0	0	0	0
0	0	0	0	0	0	0	0	0	0
0	0	0	0	0	0	0	0	0	0
0	0	0	0	0	0	0	0	0	0
1	1	1	0	0	0	0	0	0	0
0	0	0	0	0	0	0	0	0	0
1	1	1	0	0	1	1	1	1	1
0	0	0	0	0	0	0	0	0	0
0	0	0	0	0	0	0	0	0	0
0	0	0	0	0	0	0	0	0	0
0	0	0	0	0	0	0	0	0	0
0	0	0	0	0	0	0	0	0	0
0	0	0	0	0	0	0	0	0	0
0	0	0	0	0	0	0	0	0	0
0	0	0	0	0	0	0	0	0	0
0	0	0	0	0	0	0	0	0	0
1	1	1	0	0	1	1	1	1	1
0	0	0	0	0	0	0	0	0	0
0	0	0	0	0	0	0	0	0	0
0	0	0	0	0	0	0	0	0	0
0	0	0	0	0	0	0	0	0	0
0	0	0	0	0	0	0	0	0	0
0	0	0	0	0	0	0	0	0	0
0	0	0	0	0	0	0	0	0	0
0	0	0	0	0	0	0	0	0	0
0	0	0	0	0	0	0	0	0	0
0	0	0	0	0	0	0	0	0	0
1	1	1	1	1	1	1	1	1	1
1	1	1	1	1	1	1	1	1	0

0	0	0	0	0	0	0	0	0	0
0	0	0	0	0	0	0	0	0	0
0	0	0	0	0	0	0	0	0	0
1	1	1	0	0	0	0	0	0	0
1	1	1	1	1	1	1	1	1	1
0	0	0	0	0	0	0	0	0	0
1	0	0	0	0	1	1	1	1	1
0	0	0	0	0	0	0	0	0	0
0	0	0	0	0	0	0	0	0	0
0	0	0	0	0	0	0	0	0	0
0	0	0	0	0	0	0	0	0	0
1	0	0	0	0	1	1	1	1	1
0	0	0	0	0	0	0	0	0	0
0	0	0	0	0	0	0	0	0	0
0	0	0	0	0	0	0	0	0	0
0	0	0	0	0	0	0	0	0	0
1	1	1	0	0	0	0	0	0	0
1	1	1	1	1	1	1	1	1	1
0	0	0	0	0	0	0	0	0	0
0	0	0	0	0	0	0	0	0	0
0	0	0	0	0	0	0	0	0	0
1	0	1	1	1	1	1	1	1	0
0	0	0	0	0	0	0	0	0	0
0	0	0	0	0	0	0	0	0	0
0	0	0	0	0	0	0	0	0	0
0	0	0	0	0	0	0	0	0	0

30	31	32	33	34	35	36	37	38	39
1	1	1	0	0	0	0	1	1	1
1	1	1	1	1	1	1	1	1	0
0	0	0	0	0	0	0	0	0	0
1	1	1	0	0	0	0	1	1	1
1	0	0	0	0	0	0	0	1	1
0	0	0	0	0	1	1	1	1	1
0	0	0	0	0	1	1	1	1	1
0	0	0	0	0	1	1	1	1	1
0	0	0	0	0	1	1	1	1	1
0	0	0	0	0	1	1	1	1	1
0	0	0	0	0	1	1	1	1	1
0	0	0	0	0	1	1	1	1	1
0	0	0	0	0	1	1	1	1	1
0	0	0	0	0	1	1	1	1	1
0	0	0	0	0	1	1	1	1	1
1	1	0	0	0	0	0	0	0	0
0	0	0	0	0	1	1	0	0	1
0	0	0	0	0	1	1	1	1	1
0	0	0	0	0	1	1	1	1	1
1	1	1	0	0	0	0	1	1	1
0	0	0	0	0	0	0	1	1	1
0	0	0	0	0	1	1	1	1	1
1	1	1	0	0	0	0	1	1	1
0	0	0	0	0	1	1	1	1	1
0	0	0	0	0	1	1	1	1	1
0	0	0	0	0	1	1	1	1	1
1	1	1	0	0	0	0	0	1	1
0	0	0	0	0	1	1	1	1	1
0	0	0	0	0	1	1	1	1	1
0	0	0	0	0	1	1	1	1	1
0	0	0	0	0	1	1	1	1	1
0	0	0	0	0	1	1	1	1	1
0	0	0	0	0	1	1	1	1	1
1	1	1	0	0	1	1	1	1	1
0	0	0	0	0	1	1	1	1	1
0	0	0	0	0	0	0	0	0	0
0	0	0	0	0	1	1	1	1	1
1	1	1	0	0	0	0	1	1	1
0	0	1	0	0	0	0	1	1	1
1	1	1	1	0	0	0	0	1	1
0	0	0	0	0	1	1	1	1	1
0	0	1	0	0	0	0	1	1	1
0	0	0	0	0	1	1	1	1	1
0	0	0	0	0	1	1	1	1	1
1	1	1	0	0	0	0	1	1	1
0	0	0	0	0	1	1	1	1	1

0	0	0	0	0	1	1	1	1	1
1	1	1	0	0	0	0	1	1	1
1	1	1	0	0	0	0	1	1	1
0	0	0	0	0	1	1	1	1	1
0	0	0	0	0	1	1	1	1	1
0	0	1	0	0	0	0	1	1	1
0	0	1	0	0	0	0	1	1	1
0	0	0	0	0	1	1	1	1	1
1	1	1	0	0	0	0	1	1	1
0	0	0	0	0	0	0	0	0	0
0	0	0	0	0	1	1	1	1	1
0	0	0	0	0	1	1	1	1	1
0	0	1	0	0	0	0	1	1	1
0	0	0	0	0	1	1	1	1	1
0	0	0	0	0	1	1	1	1	1
0	0	0	0	0	1	1	1	1	1
0	0	0	0	0	1	1	1	1	1
0	0	0	0	0	1	1	1	1	1
0	0	0	0	0	1	1	1	1	1
0	0	0	0	0	1	1	1	1	1
0	0	0	0	0	1	1	1	1	1
0	0	0	0	0	1	1	1	1	1
0	0	1	0	0	0	0	1	1	1
0	0	0	0	0	1	1	1	1	1
0	0	0	0	0	1	1	1	1	1
0	0	0	0	0	1	1	1	1	1
0	0	0	0	0	1	1	1	1	1
0	0	0	0	0	1	1	1	1	1
1	1	1	0	0	0	0	1	1	1
0	0	0	0	0	1	1	1	1	1
0	0	0	0	0	1	1	1	1	1
0	0	0	0	0	1	1	1	1	1
0	0	0	0	0	1	1	1	1	1
0	0	0	0	0	1	1	1	1	1
0	0	1	0	0	0	0	1	1	1
0	0	1	0	0	0	0	1	1	1
0	0	0	0	0	1	0	0	0	0
0	0	0	0	0	1	1	1	1	1
0	0	0	0	0	1	1	1	1	1
0	0	0	0	0	1	1	1	1	1
0	0	0	0	0	1	1	1	1	1
1	1	1	0	0	0	0	1	1	1
0	0	0	0	0	1	1	1	1	1
0	0	0	0	0	1	1	1	1	1
0	0	0	0	0	1	1	1	1	1
0	0	0	0	0	1	1	1	1	1
0	0	0	0	0	1	1	1	1	1

0	0	0	0	0	1	1	1	1	1
0	0	1	0	0	0	0	1	1	1
0	0	0	0	0	1	1	1	1	1
0	0	0	0	0	1	1	1	1	1
0	0	0	0	0	1	1	1	1	1
0	0	0	0	0	0	0	0	0	0
0	0	0	0	0	1	1	1	1	1
0	0	0	0	0	1	1	1	1	1
0	0	0	0	0	1	1	1	1	1
0	0	0	0	0	1	1	1	1	1
0	0	0	0	0	1	1	1	1	1
0	0	0	0	0	1	1	1	1	1
0	0	0	0	0	1	1	1	1	1
0	0	0	0	0	1	1	1	1	1
0	0	0	0	0	1	1	1	1	1
0	0	0	0	0	1	1	1	1	1
0	0	0	0	0	1	1	0	0	1
0	0	0	0	0	1	1	1	1	1
1	1	1	0	0	0	0	1	1	1
0	0	0	0	0	1	1	1	1	1
0	0	0	0	0	1	1	1	1	1
0	0	0	0	0	1	1	1	1	1
0	0	0	0	0	1	1	1	1	1
1	1	1	0	0	0	0	1	1	1
0	0	0	0	0	1	1	1	1	1
0	0	0	0	0	1	1	1	1	1
0	0	0	0	0	1	1	1	1	1
0	0	0	0	0	1	1	1	1	1
0	0	0	0	0	1	1	1	1	1
1	1	1	0	0	0	0	1	1	1
1	1	1	0	0	0	0	1	1	1
0	0	0	0	0	1	1	1	1	1
0	0	0	0	0	1	1	1	1	1
0	0	0	0	0	1	1	1	1	1
0	0	0	0	0	1	1	1	1	1
0	0	1	0	0	0	0	1	1	1
1	1	1	0	0	0	0	1	1	1
0	0	0	0	0	1	1	1	1	1
0	0	0	0	0	1	1	0	0	1
0	0	0	0	0	0	0	1	1	1
1	1	1	0	0	0	0	1	1	1
1	1	1	0	0	0	0	1	1	1
0	0	0	0	0	1	1	1	1	1
1	1	1	0	0	0	0	1	1	1
0	0	0	0	0	1	1	1	1	1
0	0	0	0	0	1	1	1	1	1
0	0	0	0	0	1	1	1	1	1

0	0	0	0	0	1	1	1	1	1
0	0	0	0	0	1	1	1	1	1
0	0	0	0	0	1	1	1	1	1
1	1	1	0	0	0	0	1	1	1
0	0	0	0	0	1	1	1	1	1
0	0	0	0	0	1	1	1	1	1
0	0	0	0	0	1	1	1	1	1
0	0	0	0	0	1	1	1	1	1
1	1	1	0	0	0	0	1	1	1
1	0	1	0	0	0	0	1	1	1
0	0	0	0	0	1	1	1	1	1
0	0	0	0	0	1	1	1	1	1
0	0	0	0	0	1	1	1	1	1
0	0	0	0	0	1	1	1	1	1
1	1	0	0	0	0	0	0	0	0
0	0	0	0	0	0	0	0	0	0
0	0	0	0	0	1	1	1	1	1
0	0	0	0	0	1	1	1	1	1
0	0	1	0	0	0	0	1	1	1
0	0	0	0	0	1	1	1	1	1
0	0	0	0	0	1	1	0	0	1
0	0	0	0	0	1	1	1	1	1
0	0	0	0	0	1	1	1	1	1
1	1	1	0	0	0	0	1	1	1
0	0	0	0	0	1	1	1	1	1
0	0	0	0	0	1	1	1	1	1
1	1	1	0	0	0	0	1	1	1
1	1	1	0	0	0	0	1	1	1
1	1	1	0	0	0	0	1	1	1
0	0	0	0	0	1	1	1	1	1
0	0	0	0	0	1	1	1	1	1
0	0	0	0	0	1	1	1	1	1
0	0	0	0	0	1	1	1	1	1
0	0	0	0	0	1	1	1	1	1
0	0	0	0	0	1	1	1	1	1
0	0	0	0	0	1	1	1	1	1
1	1	1	0	0	0	0	1	1	1
0	0	1	0	0	0	0	1	1	1
0	0	0	0	0	1	1	1	1	1
1	1	1	0	0	0	0	0	1	1
0	0	0	0	0	1	1	1	1	1
0	0	0	0	0	1	1	1	1	1
0	0	0	0	0	1	1	1	1	1
1	1	1	0	0	0	0	1	1	1
0	0	0	0	0	1	1	1	1	1
1	0	1	0	0	0	0	1	1	1
0	0	0	0	0	1	1	1	1	1
1	1	1	0	0	0	0	1	1	1

0	0	0	0	0	1	1	1	1	1
0	0	0	0	0	1	1	1	1	1
0	0	0	0	0	1	1	1	1	1
1	1	0	0	0	0	0	0	1	1
0	0	0	0	0	1	1	1	1	1
0	0	0	0	0	1	1	1	1	1
0	0	0	0	0	1	1	1	1	1
0	0	0	0	0	1	1	1	1	1
0	0	0	0	0	1	1	1	1	1
0	0	0	0	0	1	1	1	1	1
0	0	0	0	0	1	1	1	1	1
0	0	0	0	0	1	1	1	1	1
0	0	0	0	0	1	1	1	1	1
0	0	0	0	0	1	1	1	1	1
0	0	0	0	0	1	1	1	1	1
0	0	0	0	0	1	1	1	1	1
0	0	0	0	0	1	1	1	1	1
0	0	0	0	0	1	1	1	1	1
0	0	0	0	0	1	1	1	1	1
0	0	0	0	0	1	1	1	1	1
0	0	0	0	0	1	1	1	1	1
1	1	1	0	0	0	0	1	1	1
0	0	0	0	0	0	0	0	0	0
0	0	0	0	0	0	0	1	1	1
0	0	0	0	0	1	1	1	1	1
0	0	1	0	0	0	0	1	1	1
0	0	0	0	0	1	1	1	1	1
0	0	0	0	0	1	1	1	1	1
0	0	0	0	0	1	1	1	1	1
0	0	0	0	0	1	1	1	1	1
0	0	0	0	0	1	1	0	0	1
1	1	1	0	0	0	0	1	1	1
0	0	0	0	0	1	1	1	1	1
0	0	0	0	0	1	1	1	1	1
0	0	0	0	0	1	1	1	1	1
0	0	1	0	0	0	0	1	1	1
0	0	0	0	0	1	1	1	1	1
1	1	1	0	0	0	0	1	1	1
0	0	0	0	0	1	1	1	1	1
0	0	0	0	0	1	1	1	1	1
0	0	0	0	0	1	1	1	1	1
0	0	0	0	0	1	1	1	1	1
1	1	1	0	0	0	0	1	1	1
0	0	0	0	0	1	1	1	1	1
1	1	1	0	0	0	0	1	1	1
0	0	0	0	0	1	1	1	1	1
0	0	0	0	0	1	1	1	1	1
0	0	0	0	0	1	1	1	1	1
0	0	0	0	0	1	1	1	1	1
1	1	1	0	0	0	0	1	1	1

0	0	0	0	0	1	1	1	1	1
0	0	0	0	0	1	1	1	1	1
0	0	0	0	0	1	1	1	1	1
0	0	0	0	0	1	1	1	1	1
0	0	0	0	0	1	1	1	1	1
0	0	0	0	0	1	1	1	1	1
0	0	0	0	0	1	1	1	1	1
0	0	0	0	0	0	0	0	0	0
0	0	1	0	0	0	0	1	1	1
0	0	0	0	0	1	1	1	1	1
0	0	0	0	0	1	1	1	1	1
1	1	1	0	0	0	0	1	1	1
0	0	0	0	0	1	1	1	1	1
0	0	0	0	0	1	1	1	1	1
0	0	0	0	0	1	1	1	1	1
0	0	0	0	0	1	1	1	1	1
0	0	0	0	0	1	1	1	1	1
1	1	1	0	0	0	0	1	1	1
0	0	0	0	0	1	1	1	1	1
0	0	0	0	0	1	1	1	1	1
0	0	0	0	0	1	1	1	1	1
0	0	0	0	0	1	1	1	1	1
0	0	0	0	0	1	1	1	1	1
0	0	0	0	0	1	1	1	1	1
0	0	0	0	0	1	1	1	1	1
0	0	0	0	0	1	1	1	1	1
0	0	0	0	0	1	1	1	1	1
1	1	1	0	0	0	0	1	0	0
0	0	0	0	0	1	1	1	1	1
0	0	0	0	0	1	1	1	1	1
0	0	0	0	0	1	1	1	1	1
0	0	0	0	0	1	1	1	1	1
0	0	0	0	0	1	1	1	1	1
0	0	0	0	0	1	1	1	1	1
0	0	0	0	0	1	1	1	1	1
0	0	0	0	0	1	1	1	1	1
0	0	0	0	0	1	1	1	1	1
1	1	1	1	0	1	1	1	1	1
0	0	0	0	0	1	1	1	1	1
0	0	0	0	0	1	1	1	1	1
0	0	0	0	0	1	1	1	1	1
0	0	0	0	0	1	1	1	1	1
0	0	0	0	0	1	1	1	1	1
0	0	0	0	0	1	1	1	1	1
0	0	0	0	0	1	1	1	0	1
0	0	0	0	0	1	1	1	0	1
0	0	0	0	0	1	1	1	1	1
0	0	0	0	0	1	1	1	1	1
1	1	1	1	0	0	0	1	1	1
0	0	1	0	0	0	0	1	1	1

0	0	0	0	0	1	1	1	1	1
0	0	0	0	0	1	1	1	1	1
0	0	0	0	0	1	1	1	1	1
0	0	0	0	0	1	1	0	0	1
1	1	1	0	0	0	0	1	1	1
0	0	0	0	0	1	1	1	1	1
1	1	1	0	0	0	0	1	1	1
0	0	0	0	0	1	1	1	1	1
0	0	0	0	0	1	1	1	1	1
0	0	0	0	0	1	1	1	1	1
0	0	0	0	0	1	1	1	1	1
1	1	1	1	0	0	0	0	1	1
0	0	0	0	0	1	1	1	1	1
0	0	0	0	0	1	1	1	1	1
0	0	0	0	0	1	1	1	1	1
0	0	0	0	0	1	1	1	1	1
0	0	0	0	0	0	0	1	1	1
1	1	1	0	0	0	0	1	1	1
0	0	0	0	0	1	1	1	1	1
0	0	0	0	0	1	1	1	1	1
0	0	0	0	0	0	0	0	0	0
0	0	1	0	0	0	0	1	1	1
0	0	0	0	0	1	1	1	1	1
0	0	0	0	0	1	1	1	1	1
0	0	0	0	0	1	1	1	1	1
0	0	0	0	0	1	1	1	1	1

40	41	42	43
1	1	1	1
0	0	0	0
0	0	0	0
1	1	1	1
1	1	1	1
1	1	1	1
1	1	1	1
1	1	1	1
1	1	1	1
1	1	1	1
1	1	1	1
1	1	1	1
1	1	1	1
1	1	1	1
1	1	1	1
0	0	0	1
1	1	1	1
1	1	1	1
1	1	1	1
0	1	1	1
1	1	1	1
1	1	1	1
1	1	1	1
1	1	1	1
1	1	1	1
1	1	0	0
1	1	1	1
1	1	1	1
1	1	1	1
1	1	1	1
1	1	0	1
1	1	1	1
1	1	1	1
1	1	1	1
1	1	1	1
0	0	0	0
1	1	1	1
1	1	1	1
1	1	1	1
1	1	1	1
1	1	1	1
1	1	1	1
1	1	1	1
1	1	1	1
0	1	1	1
1	1	1	1

1	1	1	1
1	1	1	1
0	1	1	1
1	1	1	1
1	1	1	1
1	1	1	1
1	1	1	1
1	1	1	1
1	1	1	1
0	0	0	0
1	1	1	1
1	1	1	1
1	1	1	1
1	1	1	1
1	1	1	1
1	1	1	1
1	1	1	1
1	1	1	1
1	1	1	1
1	1	1	1
1	1	1	1
1	1	1	1
1	1	1	1
1	1	1	1
1	1	1	1
1	1	1	1
1	1	1	1
1	1	1	1
1	1	1	1
1	1	1	1
1	1	1	1
1	1	1	1
1	1	1	1
1	1	1	1
1	1	1	1
1	1	1	1
0	1	1	1
0	1	1	1
1	1	1	1
0	1	1	1
1	1	1	1
1	1	1	1
1	1	1	1
1	1	1	1
1	1	1	1
1	1	1	1
1	1	1	1

1	1	1	1
1	1	1	1
1	1	1	1
1	1	1	1
1	1	1	1
0	0	0	0
1	1	1	1
1	1	1	1
1	1	1	1
1	1	1	1
1	1	1	1
1	1	1	1
1	1	1	1
1	1	1	1
1	1	1	1
1	1	1	1
1	1	1	1
1	1	1	1
1	1	1	1
1	1	1	1
1	1	1	1
1	1	1	1
1	1	1	1
0	1	1	1
1	1	1	1
1	1	1	1
1	1	1	1
1	1	1	1
1	1	1	1
1	1	1	1
1	1	1	1
1	1	1	1
1	1	1	1
1	1	1	1
1	1	1	1
1	1	1	1
1	1	1	1
1	1	1	1
1	1	1	1
1	1	1	1
1	1	1	1
0	1	1	1
1	1	1	1
0	0	0	0
1	1	1	1
1	1	1	1
1	1	1	1

1	1	1	1
1	1	1	1
1	1	1	1
1	1	1	1
1	1	1	1
1	1	1	1
1	1	1	1
1	1	1	1
1	1	1	1
1	1	1	1
1	1	1	1
1	1	1	1
1	1	1	1
1	1	1	1
0	1	1	1
0	0	0	0
1	1	1	1
1	1	1	1
1	1	1	1
1	1	1	1
1	1	1	1
1	1	1	1
1	1	1	1
0	1	1	1
1	1	1	1
1	1	1	1
1	1	1	1
1	1	1	1
0	1	1	1
1	1	1	1
1	1	1	1
1	1	1	1
1	1	1	1
1	1	1	1
1	1	1	1
1	1	1	1
1	1	1	1
1	1	1	1
1	1	1	1
1	1	1	1
1	1	1	1
1	1	1	1
1	1	1	1
1	1	1	1
1	1	1	1
1	1	1	1
1	1	1	1

1	1	1	1
1	1	1	1
1	1	1	1
1	1	1	1
1	1	1	1
1	1	1	1
1	1	1	1
1	1	1	1
1	1	1	1
1	1	1	1
1	1	1	1
1	1	1	1
1	1	1	1
1	1	1	1
1	1	1	1
1	1	1	1
1	1	1	1
1	1	1	1
1	1	1	1
1	1	1	1
1	1	1	1
0	1	1	1
0	1	1	1
1	1	1	1
0	1	1	1
1	1	1	1
1	1	1	1
1	1	1	1
1	1	1	1
1	1	1	1
1	1	1	1
1	1	1	1
1	1	1	1
1	1	1	1
1	1	1	1
1	1	1	1
1	1	1	1
1	1	1	1
1	1	1	1
1	1	1	1
1	1	1	1
1	1	1	1
1	1	1	1
1	1	1	1
1	1	1	1
1	1	1	1
1	1	1	1
1	1	1	1
1	1	1	1
0	1	1	1

1	1	1	1
1	1	1	1
1	1	1	1
1	1	1	1
1	1	1	1
1	1	1	1
1	1	1	1
1	1	1	1
1	1	1	1
1	1	1	1
1	1	1	1
1	1	1	1
1	1	1	1
1	1	1	1
1	1	1	1
1	1	1	1
1	1	1	1
0	1	1	1
1	1	1	1
1	1	1	1
1	1	1	1
1	1	1	1
1	1	1	1
1	1	1	1
1	1	1	1
1	1	1	1
1	1	1	1
0	1	1	1
0	1	1	1
1	1	1	1
1	1	1	1
1	1	1	1
1	1	1	1
1	1	1	1
1	1	1	1
1	1	1	1
1	1	1	1
1	1	1	1
1	1	1	1
1	1	1	1
1	1	1	1
1	1	1	1
1	1	1	1
1	1	1	1
1	1	1	1
1	1	1	1
1	1	1	1
1	1	1	1
1	1	1	1
1	1	1	1

1	1	1	1
1	1	1	1
1	1	1	1
1	1	1	1
0	1	1	1
1	1	1	1
1	1	1	1
1	1	1	1
1	1	1	1
1	1	1	1
1	1	1	1
1	1	1	1
1	1	1	1
1	1	1	1
1	1	1	1
1	1	1	1
1	1	1	1
1	1	1	1
1	1	1	1
1	1	1	1
0	0	1	1
1	1	1	1
1	1	1	1
1	1	1	1
0	1	1	1
1	1	1	1
